# Tracking Waste Management Information Disclosure Behavior Connected to Financial Performance through Moderating Variables

**DOI:** 10.3390/ijerph192013068

**Published:** 2022-10-11

**Authors:** Victoria Bogdan, Claudia Diana Sabău-Popa, Marcel-Ioan Boloș, Dorina-Nicoleta Popa, Mărioara Beleneși

**Affiliations:** Department of Finance and Accounting, Faculty of Economic Sciences, University of Oradea, 410087 Oradea, Romania

**Keywords:** waste information disclosure, financial performance, manufacturing industry, productivity, board size, environmental-sensitive status

## Abstract

The current challenges of a circular economy exert a high pressure on manufacturing companies that generate waste to track and implement policies to reduce them and eliminate the toxicity of residues. Hence, the purpose of this study is to analyze the waste management information disclosure linked to the financial performance of companies and test the moderating effect of internal and external variables. The average waste management information disclosure index shows a poor disclosure score for the analyzed period, however, the waste disclosure index after reaching a minimum threshold in 2019 recorded an encouraging increase at the end of 2021. Applying the fixed effects model, ordinary least squares, and two-stage least squares method, the results revealed a positive and statistically significant relationship between management information disclosure and the return on assets, while for the current ratio the connection has been invalidated. A statistically significant influence of the environmental-sensitive industry status, board size, and productivity on the moderating variables was found for the return on assets, while for current ratio, there was none. As for the alternative metrics of financial performance, the results showed that a higher degree of management information disclosure will increase the return on equity and earnings per share, while in the case of liquidity, the results are not conclusive.

## 1. Introduction

Today’s economy is an economy based on consumption, even excessive consumption, both among the population and at the level of companies. The immediate next result is an increase in waste. This reality is a worrying one because it affects people’s health, and not this alone. It is, therefore, necessary to act consciously and immediately to reduce the high volume of waste. The more that the accumulation of waste is reduced and waste is properly managed, the more the development of diseases and possible outbreaks of infections will be prevented, which will contribute to the health and well-being of people. The problem of efficient waste management is a concern at the level of company management forums because the topic of pollution and the effect of global warming on human health [1] resonates worldwide. Being energy consumers and participants in pollution, companies must strengthen their environmental management and information disclosure policies [2]. The Global Reporting Initiative through published standards supports companies to report their actions for sustainability. The GRI standards represent a sustainable performance reporting framework useful for any company as it provides multiple dimensions of environmental practices, being a good support for summarizing and scoring corporate environmental information disclosure [2]. Aspects related to waste management are provided in the specific standard 300 concerning the environment. Subdivision 306 encourages companies to disclose information on the total volume of water it discharges by quality and destination, the total weight of hazardous and non-hazardous waste and the method of disposal, significant discharges, and their impact, the transportation of hazardous waste, and the water bodies and related habitats which are significantly affected by spills and/or a runoff [3]. Waste management aims to minimize the impact on the environment and includes activities involving the prevention of waste generation [4], recycling and reuse to ensure a circular economy, optimizing the disposal process, and monitoring waste management are of interest to society and stakeholders. Thus, companies can approach a pro-disclosure behavior to legitimize their operations, reduce information asymmetry and the cost of capital, and consequently achieve a better financial performance [5]. For a good record and efficient waste management, it is important to identify the categories of waste generated by a company and hand them over to authorized collectors, for recycling, transport, treatment, or storage, as well as the monthly record and the annual reporting of the information on waste. In Romania, Government Emergency Order no. 92/2021 on the waste regime [6] was published to align national legislation with Directive (EU) 2018/851 on waste [7] to facilitate the transition to a circular economy. The regulation provides the framework for the sustainable management of materials to protect the environment, for human health, and for the rational and cautious use of resources. Certain categories of companies are required, following a waste audit, to draw up a plan/program to prevent and reduce the amount of waste and publish it on their websites. The companies that generate waste must keep a record of their management according to Government Decision no. 856/2002 regarding the record of waste management, the approval of the list containing the waste, including hazardous waste [8], and the centralized data to be transmitted annually to the environmental authorities. Government Decision no. 1.061/2008 regarding the transport of hazardous and non-hazardous waste on Romanian territory [9], Law no. 249/2015 regarding the method of managing packaging and packaging waste with subsequent transformations and additions [10], and Law no. 194/2019 regarding the selective collection of waste [11] are several other regulations with a practical incidence in Romania.

Anchored in the presented context, this study aims to track the impact of waste management information disclosure on several profitability and liquidity indicators of Romanian manufacturing listed companies for the years 2017 to 2021. Another goal was to check on the moderating effects. In this sense, three variables were picked out, an economic proxy, a governance item, and a regulatory issue. To achieve the research goals, the study employed the following questions:

**Q1.**  *Whether waste management information disclosure influence the financial performance of manufacturing listed companies?*

**Q2.**  *If economic productivity, board size, and the environmental sensitivity status of the industry may have moderating effects on the relationship between waste management information disclosure behavior and financial performance measured by profitability and liquidity indicators?*

The data and methodology used, the obtained results, and also a discussion are disclosed in this paper. To achieve coherence in the presentation, the paper is structured into six sections. After this first section, there is a short foray into the literature published on the topic, which culminated with the subsection dedicated to the research hypotheses. The third section presents the research methodology, the sample, and the supporting data of the performed tests. Further sections include the obtained results and discussion, and the last part presents the conclusions, limits, and implications of the study.

## 2. State of Art and Hypotheses Development

### 2.1. Prior Works on Environmental Information Disclosure and Corporate Performance

Waste issue is much more complex than it seems at first glance. Inadequately managed waste has a significant negative impact on the environment and people’s health, but it also represent a huge waste of resources, which are already at an accelerated rate of depletion. The GRI standard 306: Waste [12] emphasizes the relationship between materials and waste, encourages companies to report on circularity and prevention, causes organizations to recognize the responsibility for waste upstream and downstream, and recognizes the waste generated throughout the value chain. The waste component is very important in the environmental dimension of sustainability, just as the environmental dimension is an essential component of corporate social responsibility [13] because it concerns the impact of companies on living and non-living natural systems, water, land, air, and ecosystems [12]. Most research has considered environmental aspects [1,5,13,14,15,16,17,18,19,20,21,22,23,24,25,26], but few have focused their attention on waste [4,27,28].

Li et al. [1] in a study conducted on Chinese listed companies from the heavy pollution industry aimed to identify the factors that influence the quality of environmental information disclosure from the perspective of business strategy. They discover that, unlike defender-type firms, prospector-type firms reduce environmental information disclosure quality, and financing constraints mediate between corporate strategy and environmental information disclosure quality. For many researchers, measuring the impact of disclosed information about the environment on the company’s financial performance was the goal of their research [2,5,29,30,31,32,33,34,35,36,37,38,39,40,41,42,43]. Kalash in 2020 [5] performed an analysis of 66 Turkish listed companies, considering 5 years (2014–2018) to capture the effect of environmental disclosure on performance and identify the determinants of environmental disclosure. The results showed that environmental disclosure has a significant positive impact only on the operating profit margin, without presenting a significant influence on the return on equity, return on assets, and stock returns. The author also demonstrates that large companies, those with a high leverage, and companies with higher equity agency costs are willing to disclose more environmental information. Conversely, profitability, the type of industry, investment opportunities, information asymmetry, and business risk do not influence the probability that the company discloses environmental information. Investigating the particularities of Chinese companies listed for the years 2013 and 2014, Li et al. [2] looked for associations among corporate environmental performance, environmental information disclosure (measured by 38 indicators), and financial performance (evaluated by lagged ROA). The unusual results are in contradiction with most previous studies which showed that in the case of the 475 analyzed companies between corporate environmental performance and environmental disclosure, there is a U-shaped nonlinear relationship, a negative relationship between environmental disclosure and financial performance, and an insignificant relationship between environmental performance and financial performance. Focusing on energy-intensive listed Chinese companies, Li et al. [32] investigate the period of 2012–2014 and discovered that corporate environmental responsibility has a significant positive influence on business performance. The impact of corporate environmental information disclosure on the investors’ reaction was the aim of Meng and Zhang’s research in 2022 [19]. The authors determined that the accumulative abnormal returns of stocks were used to identify investors’ responses and an environmental information disclosure was evaluated according to the environmental information provided through the annual reports of all Chinese listed companies, during 2004–2020. Environmental information disclosure has a significant negative impact among investors, and companies with high institutional shareholding and heavy-polluting companies are more prone to negative reactions from investors. Examining listed Brazilian companies, Pedron et al. [34] found that environmental disclosure positively affects the value of these companies. The authors noted that the 69 companies investigated between 2006 and 2012 show significantly different characteristics between those that disclose/do not disclose environmental information. Dutta [14] analyzed 22 listed Finnish companies that published sustainability reports in the period of 2008–2015. The results revealed that those companies that have a high environmental performance in terms of water consumption and GHG emissions have their sustainability reports externally assured.

Several Romanian authors have studied the disclosure behavior of Romanian listed companies regarding non-financial information in general [44,45,46,47,48] and environmental disclosure in particular [24,25,26,42]. In terms of disclosed non-financial information, Sava et al. [44] investigates large Romanian companies and finds that only about a quarter of them published non-financial information on their website in 2017. Tiron-Tudor et al. [45] observed a slight increase in the level of disclosure of non-financial information among listed Romanian companies after the implementation of the European Directive 2014/95 [49]. The concentration and awareness of ESG issues manifested itself the most within energy companies, where the growth was more considerable. Milu and Hategan [47] identified and analyzed all the companies to which the directive applies and found that the compliance degree with the reporting requirements is still uncertain and only the listed companies are concerned with improving reporting. Romanian banks published non-financial information before the implementation of the directive (CSR reports have been available since 2009) according to Tachiciu et al. [46], but corporate responsibility policies are decoupled from the risks associated with the business model. Having as test subjects 63 state-controlled enterprises in Romania, Dragomir et al. [48] obtains that a non-financial reporting quality score is positively correlated with the company size, corporate governance score, monopolistic position, environmental impact, and the state’s strategic objectives, but negatively correlated with the concentration of ownership. Analyzing the content of different types of reports and statements published by Romanian companies in the period of 2006–2008, Ienciu et al. [24] observed that a low level of environmental information was disclosed, but it increased from one period to another. Unlike Romanian companies, Hungarian companies from the same sector of activity (heavy polluting industries) present a higher qualitative level for environmental reporting. Ienciu [25] continues to study the level of environmental disclosure in the annual reports of the 64 Romanian companies listed up to the year 2010. However, the conclusion remains that the reported environmental information suffers from irrelevancy and incompleteness. The size of the company, the stock market sector, and the percentage of export sales are factors that influence the variation in environmental reporting. Istrate et al. [42] analyzed the annual reporting documents of the 65 Romanian listed companies and found a significant influence of environmental information on their global performance in 2013 compared to 2011. The research of Dinca et al. [26] was carried out during 2013–2017 and targeted 100 Romanian listed companies with the highest pollution risks. The authors find that the size of the company evaluated according to the number of employees and financial profitability, positively influences the disclosure of environmental information. However, indebtedness degree and the entity’s age have no influence. Just like Ienciu et al. [24], Ienciu [25] and Dinca et al. [26] note the low level of environmental disclosure in the case of Romanian companies. Aspects related to environmental reporting and disclosure were also treated by Oncioiu et al. [50] in the context of the simultaneous approach to the subjects of corporate sustainability reporting and financial performance. Based on the 320 questionnaires applied to Romanian managers, the authors identify positive correlations between corporate sustainability reporting and the level of financial performance and consider this way of reporting useful to managers in their environmental decision-making process.

### 2.2. Hypotheses

**Figure 1:**
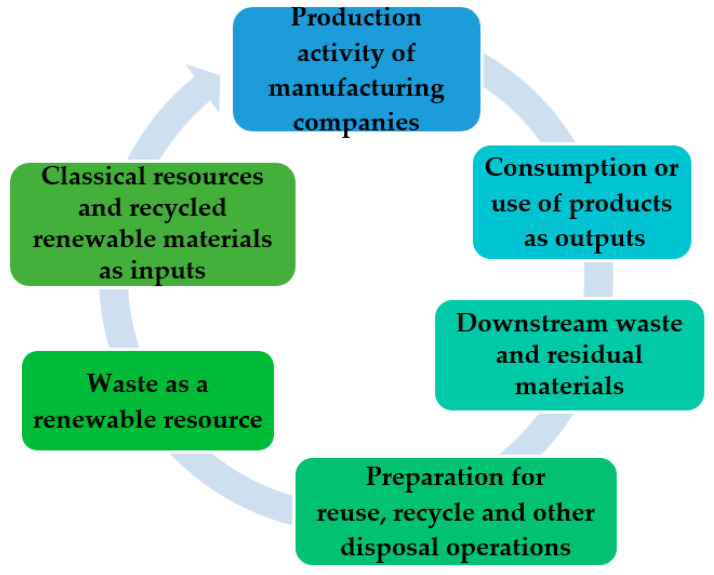
Waste flow in the manufacturing industry.

Environmental information disclosed by companies refers to the presentation and disclosure of information regarding environment protection and the natural resources used and has a major influence on society by contributing to the improvement of the company’s image and enhancing the quality of financial and non-financial reporting [51]. In the structure of the environmental information required to be disclosed based on the GRI standards, one of the most current but also controversial categories is that of waste information disclosure, so that the resource recycling process of companies is optimized related to the strategic objectives of sustainable business development. This study closely followed the waste disclosure requirements as presented by the *GRI standard 306: Waste* [12] to develop the hypotheses and design the research framework. In the process of producing goods or providing services, companies can generate waste as it can also result from activities upstream and downstream of the company’s main activity. To achieve the efficient management of waste and residual materials, it is vital to understand the process of using and recycling resources within the production activity of manufacturing companies, as well as the implications of the stages of this circuit on all parties involved (Figure 1). The management of materials and waste is a problem that encompasses at the level of the business’ organization of different units and departments, as well as operational cycles, and from the perspective of resources flow, various organizations and companies interact in their handling to recycle the materials resulting from the stages of the resource recovery process [27]. The objective of the reporting company is that replacing these primary resources with waste leads to a safe path to sustainable development [28].

Currently, the interest in studies related to waste information disclosure in correlation with various indicators of business development is registering a significant increase. Thus, this work intended to look upon the main influences of the waste information disclosure behavior of manufacturing listed companies on financial performance and analyze the moderating impact of annual productivity, board size, and the environmental-sensitive industry. According to the legitimacy theory, the more a company discloses and reports on detailed environmental information, the more it will improve its degree of legitimacy and, at the same time, its image amongst the public [13,15,52]. Therefore, to ensure a high quality of environmental information disclosure as well as financial performance, the distinct and detailed tracking of reported information is necessary to have an effective control over pollution, the resulting waste, and other environmental information. To formulate the working hypotheses, this study aimed to identify former studies’ key results on the nexus between waste information disclosure and financial performance, designed on the foundation provided by stakeholders, legitimacy, signaling, voluntary disclosure, and resource-based theories. As Guo et al. [13] pointed out, the disclosure of environmental information influences financial performance through the pressure exerted by the external environment but also by internal governance policies. Companies, to obtain a high environmental performance, must manage waste effectively, prevent massive and aggressive pollution, and reduce pollution emissions. The actions taken to protect the environment, however, generate high operational costs that affect the performance of companies [17]. Consequently, if companies carry out activities with a high degree of pollution, their financial performance can be significantly affected by the additional costs generated by the management of environmental issues [35].

Prior studies on the topic can be grouped into three main categories: those that demonstrate a positive correlation, ones that, on the contrary, proved a negative influence of environmental information disclosure on profitability, and those that argued that the influence is not relevant. On one hand, studies [15,29,36,53] that showed the positive influence of environmental information disclosure on a company’s image, reputation, a reduction in financing costs, and an increase in its profitability are the ones that placed the theoretical framework of the research on the pillars of signaling theory. Thus, the quality of reporting and environmental information disclosure is a responsibility towards society and is part of the company’s global performance concept, and through the amount and degree of detailed information disclosure, companies send signals to the market and compete with other companies in the same sector. On the other hand, several works [2,37,40,41,54,55] emphasized that under the pressure exerted by different stakeholders, companies trying to satisfy their requirements can develop a speculative behavior that leads to a decrease in responsibility towards the environment and an increase in environmental protection costs, which negatively affects profitability and liquidity [20,38]. Yet, there are also studies [30,36,56] that claim no significant impact of environmental information disclosure on financial performance indicators; the benefits are undoubted on the company’s image and the strengthening of shareholders’ trust.

Therefore, the following hypotheses were issued:

**Hypothesis** **1** **(H1).** *Waste management information disclosure has a positive and significant influence on financial performance measured by profitability indicators (ROA, ROE, EPS).*

**H1a.**  *Waste management information disclosure has a positive and significant influence on ROA.*

**H1b.**  *Waste management information disclosure has a positive and significant influence on ROE.*

**H1c.**  *Waste management information disclosure has a positive and significant influence on EPS.*

**Hypothesis** **2** **(H2).** *Waste management information disclosure has a positive and significant influence on financial performance measured by liquidity indicators (SOL and CR).*

**H2a.**  *Waste management information disclosure has a positive and significant influence on SOL.*

**H2b.**  *Waste management information disclosure has a positive and significant influence on CR.*

Additionally, current works have focused on the analysis of the moderation and mediation effects generated by different indicators of economic development or corporate governance, offering the possibility of more stratified and in-depth analyzes. According to Qui et al. [36], profitable companies have the resources to invest in stakeholder engagement practices, especially about employees, so an effective collaboration and communication with these key stakeholders can facilitate building a solid reputation and increase the credibility of a company, that can lead to the reduction in costs and agency conflicts. Tian et al. [21] and Yang et al. [38] used environmental regulation as moderating variables, while Li and Xiao [16], but also Yang et al. [38], followed a methodology on board executive compensation as a metric for internal stakeholders. The bonuses, incentives, and benefits received by executive directors to achieve the performance indicators can greatly influence the disclosure degree of environmental information, and the quantity, quality, and fairness of the reported items. Thus, Yang et al. [38] use the natural logarithm to test the moderation effect of global compensation of the top three best-paid directors. The present study considered the annual economic productivity, board size, and environmental-sensitive industry as moderating variables that impact the intensity of the relationship between waste information disclosure and financial performance.

Consequently, we developed the following hypotheses:

**Hypothesis** **3** **(H3).** *Economic productivity has a moderating effect on the relationship between waste management information disclosure and financial performance.*

**Hypothesis** **4** **(H4).** *The status of environmental sensitivity of the industry moderates the relationship between waste management information disclosure and financial performance.*

**Hypothesis** **5** **(H5).** *Board size moderates the relationship between waste management information disclosure and financial performance.*

## 3. Materials and Methods

The paper aims to analyze how the waste management information disclosure behavior of manufacturing listed companies influences financial performance, with particular attention to the moderating effects. Twenty-eight Romanian manufacturing listed companies were examined, covering the period of 2017–2021. In the first stage of the selection process, all companies in the manufacturing industry listed on the BSE (Bucharest Stock Exchange) were included in the analysis. These were investigated according to the nature of their activities. In the second stage, the manufacturing companies whose CANE (Classification and Coding of Activities in the National Economy) classification code is registered in class two were included in the sample. Thus, the sampled companies are grouped into 21 categories according to their CANE classification code, as can be seen in Table 1.

Data gathered included two categories: financial and non-financial information. All data were collected manually from the annual reports of companies. To collect information on companies’ size, we have analyzed and collected data on turnover and the number of employees. To measure financial performance, the following profitability indicators were selected: EPS (earnings per share), ROE (return on equity), ROA (return on assets), and liquidity indicators: SOL (solvability) and CR (current ration). As a control variable, LEV (leverage) was used as moderating the variables of productivity, board size, and the environmental-sensitive industry. Data on companies’ board size was also collected manually from the annual reports. From the environmental items, the present study focused on finding data on waste. The information was tracked by reading the non-financial statement/declaration included in the annual reports or, where such a report was not found in the content of the annual report, the information was gathered from reading other environmental information presented in the sustainability report of selected companies. Thus, using the scoring method, information disclosed on how the company is involved in the prevention of obtaining waste and how it manages the waste that cannot be avoided as a result of the manufacturing processes, but also of the operations that take place upstream and downstream of the production cycle [12], were noted. To calculate a waste management information disclosure index on average (*WMnID*), the following scores were assigned: 0 for no information disclosed; 1 for less and poor information; 2 in the case of satisfactory information being disclosed but not presented in detail; and 3 in the case of rich and detailed information being disclosed. Table 2 present the variables and their description.

As mentioned previously, several studies support the existence of a positive influence between environmental information disclosure (EID) and financial performance (FP), as well as other studies demonstrate the opposite, while others argued that the connection is not relevant [36,37,53]. Therefore, in this study, to capture the manufacturing listed companies’ financial performance, five proxies were used: ROA, ROE, EPS, SOL, and CR. The return on assets (ROA) was used to gauge the financial performance as a profitability indicator, while the current ratio (CR) was a liquidity indicator. The ROE, EPS, and SOL were used to implement robustness tests. We selected these indicators based on the fact that they are classical accounting-based indicators; ROA and ROE are frequently used to evaluate financial performance [57].

As moderating variables in explaining the strength of the relationship between waste management information disclosure and financial performance, the potential influence of average annual productivity, board size, and the environmental-sensitive industry status have been tested. The awareness of environmental protection at the level of Romanian companies still registers a rather low level [58]. The environmental behavior of the companies is largely determined by compliance needs with the environmental regulations and practices and is mostly influenced by financial aspects related to the costs involved. The same report, however, emphasizes that, in recent years, there have been positive signals from companies to improve the situation by identifying new ways to manage waste as efficiently as possible and introducing the mandatory internal auditing of waste management practices. By adopting environmental strategies, Romanian companies are more and more interested in improving environmental awareness. The industry has been suggested as a factor that affects environmental and financial performance [33]. Heavily polluting companies are under more institutional pressures and must increase investments in environmental protection. Previous studies showed that industry differentiation moderates corporate social responsibility and financial performance [59]. The classification of environmentally sensitive industries was made on those outlined by Wang et al. [39] and is based on the existing green regulations in the field of environmental protection and the inclusion of the sampled companies in this category was made according to the CANE classification code. As it is well known, companies from high-polluting industries feel a constant pressure from society and regulatory institutions to increase investments in the field of environmental protection. At the same time, the industry is a factor that may affect both ESG disclosures and financial performance [33,59,60]. Analyzing from the perspective of Romanian companies with over 500 employees, the implementation of circular economy principles, Hategan et al. [61] confirmed the correlation between the non-financial reporting indicators and financial performance; the positive correlations between the non-financial reporting score, ROE, and ROA indicators have been demonstrated for the manufacturing industry. For these reasons, this study tested whether the environmentally sensitive industry status exhibits a moderating effect on waste information disclosure and financial performance. Moreover, several previous studies [62,63,64,65,66,67] have shown that the size and structure of the board of directors influence the financial performance of companies. So, another goal was to test whether the number of directors on the board exerts a moderating effect between waste management information disclosure and financial performance. Finally, the investigation focused on testing if the average productivity moderates the relationship between waste management information disclosure and financial performance. This reasoning started from the fact that when businesses disclose carbon aspects, they may tend to disclose more information related to financial performance, as economic benefits with low carbon emissions, as we found in the work of Yuan and Pan [68]. This may lead to the improvement of the total factor of the enterprise productivity through high-quality monetary carbon information disclosure and the gain of a competitive market advantage [68].

As control variables, the study used variables already confirmed as having an impact on financial performance [23], financial leverage, firm size proxied by turnover and the average number of employees, and the quality of audit reports (Big4) [31,39]. Wang et al. [39] mentioned that when compared to smaller businesses, larger ones have an easier time outperforming the competition due to their larger resource bases and competitive advantages [69]. The public’s focus on large companies increases the pressure on these businesses to adopt EID and improve their financial performance. The number of employees and the turnover are measures of a company’s size. Leverage is a measure of a company’s exposure to financial risk and can sway the actions of those with a special interest in its success [22]. Corporations may easily be brought down by monetary constraints. High levels of financial leverage increase a company’s risk of losing the market share, which can harm the company’s bottom line, growth prospects, and overall worth [22,39]. In the meantime, the quality of financial reporting and ESG disclosure influences financial performance, as shown in Aldamen et al. [70], Farcane et al. [71], and Wang et al. [39]. This paper considered that the audit report prepared by one of the Big Four companies for the investigated companies can be the variable that indicates the quality of financial reporting. Therefore, a score of 1 was awarded if the company’s annual financial statements were audited by one of the Big Four and a score of 0 if they were audited by others.

**Figure 2:**
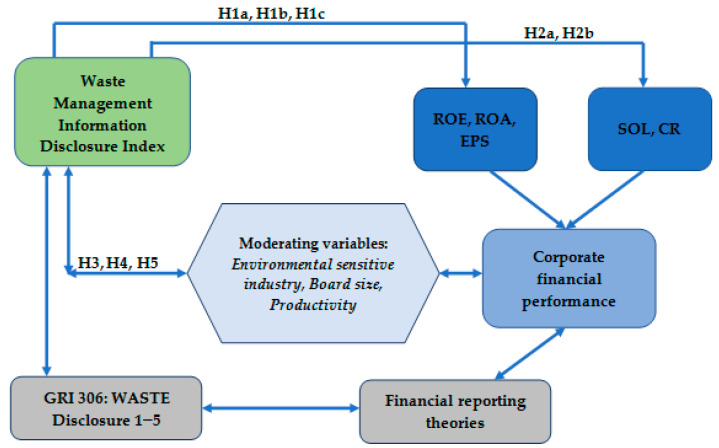
Theoretical research framework.

Synthetically, the research framework is presented as follows (Figure 2).

To capture the relationship between waste management information disclosure and a company’s financial performance, this work used initially, as a proxy, the return on assets (ROA) as a profitability indicator together with the current ratio (CR) as a liquidity indicator. Starting from the observation of Xia and Wang [40], Yang et al. [38], and Wang et al. [39], to examine the impact of waste management information disclosure behavior on financial performance, the analysis started from the following model:

(1) $$financia{l_{perf}}_{it}=\beta_{0}+\beta_{1}\times WMnID_{it}+\beta_{2}\times Size_{it}+\beta_{3}\times LEV_{it}+\overset{2021}{\underset{t=2017}{\sum }}\gamma_{t}\times {\left(year\right)}_{t}+\varepsilon_{it}$$

where $WMnID_{it}$ is the waste management information disclosure index, Size is company size proxied by the average number of employees or turnover, LEV is the financial leverage, $\beta_{i}$, *i* = 1...3 are the parameters of the model, $\gamma_{t}\;$ are the coefficients of the dummy year variables, and $\varepsilon_{it}$ is the model residual.

Furthermore, a model was developed (1) capturing the moderating effect of the environmental-sensitive industry status, board size, and productivity on waste management information disclosure and financial performance:

(2) $$financia{l_{perf}}_{it}=\beta_{0}+\beta_{1}\times WMnID_{it}+\beta_{2}\times Size_{it}+\beta_{3}\times LEV_{it}+\beta_{4}\times WMnID\times envir_{sensitive}{}_{it}+\overset{2021}{\underset{t=2017}{\sum }}\gamma_{t}\times {\left(year\right)}_{t}+\varepsilon_{it}$$

(3) $$financia{l_{perf}}_{it}=\beta_{0}+\beta_{1}\times WMnID_{it}+\beta_{2}\times Size_{it}+\beta_{3}\times LEV_{it}+\beta_{4}\times WMnID\times board\_size_{it}+\overset{2021}{\underset{t=2017}{\sum }}\gamma_{t}\times {\left(year\right)}_{t}+\varepsilon_{it}$$

(4) $$financia{l_{perf}}_{it}=\beta_{0}+\beta_{1}\times WMnID_{it}+\beta_{2}\times Size_{it}+\beta_{3}\times LEV_{it}+\beta_{4}\times WMnID\times PRD_{it}+\overset{2021}{\underset{t=2017}{\sum }}\gamma_{t}\times {\left(year\right)}_{t}+\varepsilon_{it}$$

where $WMnID_{it}$ is the waste management information disclosure index and *WMnID*×envir_sensitive, *WMnID*×BS, or *WMnID*×PRD are the interaction terms capturing the moderating effect on the relationship between the waste information disclosure and financial performance, $\beta_{i}$, *i* = 1…4 are the parameters of the model, $\gamma_{t}$ are the coefficients of the dummy year variables, and $\varepsilon_{it}$ is the model residual.

The study continued to test the multicollinearity, which considered the high degree of correlation between the independent variables that can distort the regression results, according to Pallant [72]. Essential information about the presence of multicollinearity can be found in the correlation matrix and the variance inflationary factor (VIF). For the correct analysis of panel data in multiple regression, heteroskedasticity must be followed because, if not, this can lead to the invalidation of statistical results [73,74]. As a consequence, the Breusch and Pagan LM [75] test was applied to detect the heteroskedasticity and normality of the residuals. Additionally, to eliminate the problem of correlated error items, the autocorrelation test was applied to the panel data to find serial or first-order autocorrelation. The correlation between the residuals and items, known as the cross-sectional dependence, was also examined with the help of the Breusch and Pagan test. After that, the Hausman test along with the redundant fixed effects LR test was used to determine whether the fixed or random effects model is appropriate for this study. Heteroskedasticity adjusted standard errors based on improving the standard errors of the estimators without changing the coefficient values which were used to treat cross-sectional heteroskedasticity, and the Durbin–Watson statistics were used to check for residual autocorrelation. Using an adjusted R2, RMSE and the standard error of the model, the goodness of fit of the models has been evaluated, while the validity of models has been checked with the Fisher test. The E-Views 12 software package was used to estimate the proposed econometric models. As a robustness test, the potential endogeneity was carefully treated as well as the alternative testing measures of a company’s financial performance. The two-stage least squares (2SLS) method was employed to address the endogeneity issue. *WMnID*, with one lag period (*WMnID*) which is used as an instrumental variable, was utilized to estimate the two-stage least squares model. It is generally accepted that a company’s financial performance in the current period would not be influenced by the lag WMnI, while the lag WMnI would affect the current period WMnI because the variable is classically considered as a variable with inertia [39]. Additionally, Roberts [76] suggested, as a robustness check and endogeneity approach, a time lag between measures of the explanatory factor (*WMnID*) and financial performance (ROA, ROE, EPS, SOL, CR), which is necessary due to the dynamic characteristic of information disclosure and the fact that financial performance might relate primarily to the former information disclosure [18]. The endogeneity problems were found not to be serious, based on the Hausman specification test, so the ordinary least squares (OLS) method was found to be more efficient than the instrumental variables method. Hence, initially, the study employed the return on assets (ROA) and current ratio (CR) as metrics for financial performance, and to test the robustness of the results, additional proxies were used, such as EPS, SOL, and ROE, since ROA and ROE are frequently employed to evaluate a company’s financial performance [57].

## 4. Results

**Figure 3:**
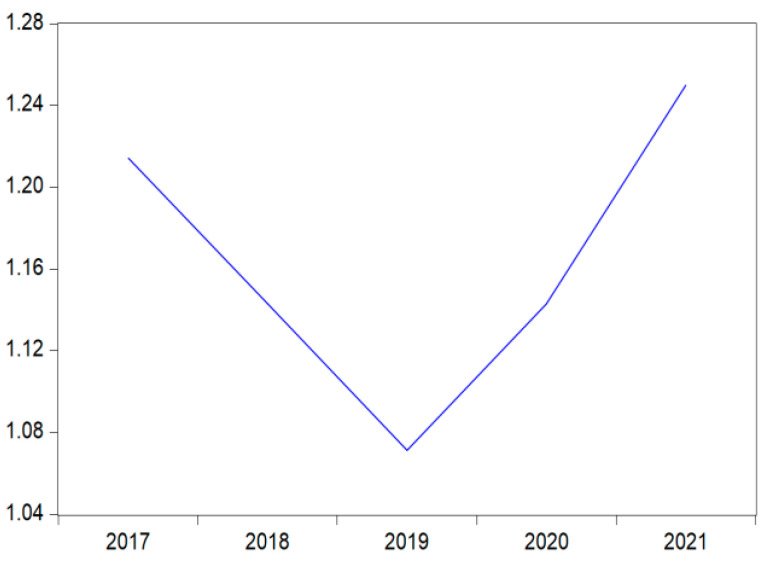
Waste management information disclosure index for the period 2017–2021.

The descriptive analysis results presented in Table 3 indicate that the average value of waste information disclosure for the total observations was 1.164, the average value showing that companies disclose less and poorer information on waste management, except for a few companies which present quantitatively more information. However, the quality of the waste disclosure index has decreased in 2019, registering a minimum value of (1.07), and then it begins to slowly increase, achieving a value of 1.25 at the end of 2021 (Figure 3). The average value of a board size is 4.58, with a maximum value of 13 members, while the average number of employees is almost 515 individuals, and the financial leverage rate is −5.3%. Approximately 64.3% of companies belong to heavy pollution industries and only 28.6% of companies are audited by the Big Four (Table 3).

**Figure 4:**
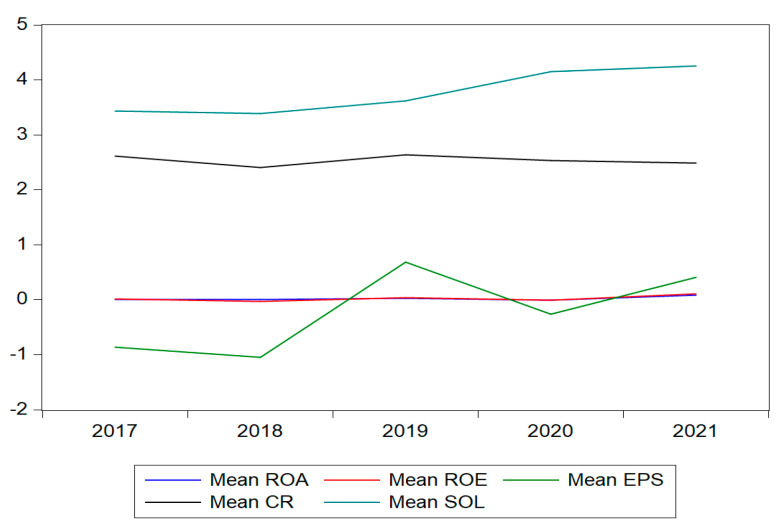
The trend of financial performance indicators.

All financial indicators exhibit large ranges, showing that there is a certain difference in the profitability of companies. From 2017 to 2021, solvability and earnings per share registered an upward trend, while the return of equity and return on assets showed an almost linear pattern, reaching a mean value of 0.023 (ROA) and 0.027 (ROE) (Table 3). The ROA value ranges from −0.81 to 1.256, and the standard deviation is 0.162, indicating that there is a certain difference in the profitability of companies. This conclusion is maintained also for the other financial indicators such as ROE, SOL, CR, or EPS. This looks like companies have not achieved good results in terms of increasing profits and saving financial resources, and their performance is relatively poor (Figure 4). The probability of Jarque–Bera test for assessing the normality of the distribution revealed that the data is not normally distributed and the probability of the statistical test is 0. Statistically, two numerical measures of shape—skewness and excess kurtosis—can be used to test for normality. If skewness is not close to zero, then the data set is not normally distributed. In this case, the data are characterized by asymmetry. The kurtosis value is far from the expected value of 3 and positive values indicated a “heavy-tailed” distribution.

The empirical results supported a positive and statistically significant connection between waste information disclosure and company size proxied by the average number of employees, supporting the literature aforementioned: that larger companies have more resources and bigger competitive advantages and more easily achieve a better performance [69]. Pearson’s correlation coefficients are reported in Table 4. Large companies attract more attention from the public, but also more pressure to implement environmental disclosure and achieve a better financial performance [39]. Nonetheless, these outcomes only prove that the pairwise correlations and multiple regression analysis may generate different results. The particular high correlation coefficients of the turnover, productivity, and the number of employees reside in the fact that the productivity is computed based on these indicators, therefore we will use these indicators by turn to avoid multicollinearity.

The correlation matrix (Table 4) also shows that the correlation coefficients between the independent, mediating, and control variables are much lower than the threshold of 0.60, pointing out that the multicollinearity problem is not serious [23]. Additionally, the variance expansion factor of the basic model is less than 2 (Table 5), showing that there is no multi-collinearity.

## 5. Discussion

### 5.1. Analysis of the Moderation Effects

In the first stage of the analysis, the ordinary least squares (OLS) method was used for a cross-section of all estimations. Testing of the redundant fixed effects was used to determine which of these models is suitable for modeling the data set (fixed effects, periodic effects, cross-sectional effects, or both). As the probability of the test is less than 0.05, it implies that the effects are statistically significant at a 5% level; therefore the fixed effects model (FEM) is suitable for estimations. Additionally, the Hausman test confirmed the same conclusion; the low probability of the Hausman test suggested the use of FEM. Therefore, the fixed effects model is used for testing, which reduces the endogeneity of the model [38]. Table 6 provides the results of the fixed effects regression analysis for two benchmark variables of financial performance, the ROA as a profitability indicator and the CR as a liquidity indicator. For each of those variables, six different models have been estimated, as follows: models 1 and 7 include the impact of the core independent variable–waste disclosure index together with the control variables on both core financial proxies (ROA and CR), the empirical results indicating a positive and statistically significant relationship only between the waste information disclosure and ROA indicator as the profitability proxy, confirming in this way the hypothesis H1a. For the case of the liquidity indicator (CR), the relationship has been disproved, invalidating hypothesis H2b. With the control variables, the results are oscillating. The board size negatively impacted the liquidity of the companies, while turnover positively affected only the profitability of the companies captured by the ROA. The number of employees has proved to be robust, positively affecting both the profitability and liquidity of the companies, captured through the ROA and CR. The impact of financial leverage on company performance is positively related to profitability, but negatively related to liquidity.

Models M2–M4 and M6–M8 captured the impact of the moderating effects on both proxies of financial performance. To design the models, this work followed the analyzes carried out by Luo et al. [43], Wang et al. [39], Xia and Wang [40], Pedron et al. [34], and Yang et al. [38]. The regression results of the moderating effects (Table 6) show a statistically significant impact for all three interaction terms in the case of the ROA, confirming the hypotheses H3, H4, and H5, while for the CR indicator, the results signal a lack of statistical significance, invalidating the hypotheses. The regression coefficient of *Envir_sensitive* × *WMnID* is significantly positive at the level of 1% in model M2, indicating that the performance of companies who disclose information about waste is higher. With the strengthening of waste disclosure, the more waste information a company discloses, the higher impact it will have on their FP, confirming H4. The results supported the hypothesis that when the status of the environmental-sensitive industry is taken into account as a moderating variable, the higher the environmental-sensitive industry, the stronger the positive correlation between the waste disclosure and financial performance. In models M3 and M4, the regression coefficients of *Board size* × *WMnID and PRD* × *WMnID* are significantly negative at the 1% level, indicating that when companies face a strong productivity or have a larger board, the negative relationship between the quality of the waste disclosure and financial performance is deepened, and H3 and H5 are verified. The results highlighted a positive impact of the average waste management information disclosure score on financial performance, while the interaction term between the waste management information disclosure index and productivity is negative and statistically significant, revealing that an increase in the disclosure index could lead to a decrease in the financial performance when the productivity increases. Therefore, when the board size, as well as the productivity, have been added to the model as moderating variables, with the increase in the board size or productivity level, the negative impact of waste disclosure on profitability is deepened. In the case of the liquidity indicator (CR), all three regression coefficients of the interaction terms were not significant; that is, board size, productivity, or the status of the environmental-sensitive industry did not have a moderating role in the relationship between the quality of waste information disclosure and the financial performance, rejecting the hypothesis H3–H5.

In most of the models estimated for both financial performance indicators (the ROA and CR), the waste management information disclosure is associated with a higher profitability and liquidity. Regarding the significance of the control variables, the board size, firm size (turnover and the number of employees), or leverage exhibited in most cases had a significant impact on the financial performance of companies. However, board size pointed out a negative impact revealing that a higher board will lead to a decrease in financial performance. The effect of company size on financial performance in all of the cases is positive and significant for both measures of performance. Financial leverage, however, has an inconclusive sign, being either positively related to profitability as well as negatively related to liquidity. Financial leverage can positively affect company performance because leverage may be seen as a tool for disciplining management. As such a positive relationship is awaited based on the agency theory. Results showed that financial leverage has a positive impact on performance if the total amount of debt does not exceed the amount of equity. However, this is not always valid for companies with too much debt. This is because a high leverage can lead to significant financial constraints and negatively impact performance. Positive leverage occurs when a business borrows funds and then invests the funds at a higher interest rate than the rate at which they were borrowed. However, leverage can become negative if the rate of the return on invested funds falls, or if the interest rate on borrowed funds increases. Lestari [77] proved that the debt ratio positively impacts the return on assets and return on equity. The debt-equity ratio has a positive influence on the return on assets but has a negative and significant effect on the return on equity and shareholder profits. Regarding the hypotheses on residuals, the potential econometric problems of heteroscedasticity and cross-sectional dependence are found in the data. To solve these, we have applied OLS with heteroscedastic panels and corrected standard errors (OLS-cross section PCSE). Molla et al. [74] found a strong reason for the estimator option and discussions in Bailey and Katz [78] and Hasan et al. [79], who directed this analysis towards the PCSE estimation which is robust both in terms of unitary heteroscedasticity and against the potential correlations between items.

### 5.2. Robustness Analysis

#### 5.2.1. Endogeneity

The two-stage least squares (2SLS) method was employed to approach endogeneity. *WMnID* with one lag period (*LWMnID*), an instrumental variable, was utilized to estimate the two-stage least squares model. It is generally agreed that company financial performance in the current period would not be influenced by the lag *WMnID*, while the lag *WMnID* would affect the current period *WMnID*, since *WMnID* is traditionally considered as an inertial variable.

#### 5.2.2. Alternative Measures of Financial Performance

To ensure that the benchmark results were not affected by other indicators for measuring corporate financial performance, this study used ROE, SOL, and EPS as alternative measures. The results showed that the research findings were solid. To solve the problem of endogeneity, a two-stage least square (2SLS) regression models M1′–M12′ was used (Table 7) for all three alternative proxies of financial performance (SOL, EPS, and ROE). The empirical results pointed out that the significant impact of the waste management disclosure index on the financial performance proxied by ROE and EPS validates the hypotheses H1b and H1c as well as on the solvability validating partially the hypothesis H2a. The results are robust and it preserved in almost all the cases. Therefore, for the ROE and EPS, which captured the profitability of the companies, the impact is positive and statistically significant, revealing that a higher degree of disclosure regarding waste management information will increase the profitability of the companies, while in the case of the liquidity, the results are inconclusive, only one model revealing a positive and statistically significant impact. Regarding solvability, the impact is rather negative and robust from the perspective of statistical significance, revealing that most likely a higher level of waste disclosure will lead to a decline in the solvability of the companies. The board size exhibited a negative influence on the financial performance and the results preserved this in all models. The productivity positively impacted the financial performance for all proxies, revealing a statistically significant effect in all models. The impact of turnover is positive and statistically significant in all models, while control variables such as the number of employees or financial leverage exhibited an inconclusive impact suffering from the lack of statistical significance.

The moderating role of the environmental-sensitive status of the industry on the relationship between waste management information disclosure and financial performance, proved to be positive for all profitability indicators (ROA, ROE, EPS), revealing that in these industries, a higher degree of disclosure increases the profitability of the companies, while the results suffer from a lack of statistical significance in the case of liquidity indicators. Therefore, hypothesis H4 is only partially confirmed rather by the moderating effect on profitability. The moderating effect of board size is statistically significant and negative for the profitability indicators (ROA, ROE, and EPS) and positive for solvability (SOL). In the case of current liquidity, the impact has not been found statistically significant. Therefore, we can conclude that in the majority of the cases, hypothesis H5 has been validated. The moderating effect of economic productivity is statistically significant and negative for the profitability indicators (ROA, ROE, and EPS) as well as for solvability (SOL). In the case of current liquidity, the impact has not been found statistically significant. Therefore, we can conclude that in the majority of cases, hypothesis H3 has been validated.

The *WMnID* has a positive impact on financial performance, which means that the more waste information is disclosed, the better the FP of the company. This is in line with the results of Liu et al. [80] and Wang et al. [39], who found that the positive effect of the EID on companies’ FP is relevant and in contradiction to the findings of Xia and Wang [40] and Aragon-Correa et al. [81], who proved a negative impact. Wang et al. [39] explained this from the perspective of the voluntary disclosure theory, pointing out that companies disclose more green information to obtain more economic benefits than to respond to institutional pressure [82]. This is consistent with prior studies [31,83,84] that reveal that a higher degree of environmental information disclosure is correlated with improved company performance, yet these results are contrary to the findings of Qiu et al. [36] and Liu and Zhang [85], who argued that there is no or a negative correlation between environmental information disclosure and financial performance. After conducting the moderation analysis, we can emphasize that compared to similar studies [34,38,39,40,43,61] conducted on environmental information disclosure and corporate performance, the results are more nuanced and indicate mixed influences, which leads us to the resolution of more detailed and in-depth research in the future, on a more generous sample and a larger and more complex data set.

## 6. Conclusions

The core of the present empirical study is to track waste management information disclosure behavior related to financial performance and capture the moderating effects of three different variables. As far as we know, such a study that follows and analyzes the waste component within the framework of environmental information disclosure at the level of manufacturing private companies has not previously been carried out in Romania, however, works on environmental issues connected to corporate performance are continuously increasing from one period to another. Due to the multiple challenges and opportunities that the circular economy implies, it can be noticed that the present study adds value from the perspective of the examination, analysis, and results obtained on this niche of waste management information disclosure connected to financial performance. Hence, it was found that the average waste management information disclosure index is 1.164, which shows a poor disclosure score for the entire period, however, the waste disclosure index after reaching a minimum threshold in 2019, recorded an encouraging increase in the value of 1.25 at the end of 2021. The financial performance of the analyzed companies did not record significant changes over the period; the evolution of the analyzed indicators (the ROA, ROE, EPS, SOL, and CR) on average indicates a poor performance of the companies.

Applying the FEM, the results led us to the findings of a positive and statistically significant relationship only between the waste information disclosure and ROA, while for the CR, the connection has been disproved. This finding means that the influence of waste management information disclosure on financial performance must be stratified by investigating the impact of disclosure behavior on several categories of performance indicators. It also confirms the mixed results provided by previous studies and invites analysis in layers based on the influences exerted on the interests of different stakeholders and the impact on society. Pressures from the external environment of manufacturing companies as well as from internal governance policies determine the influence of *WMnID* on financial performance. For these reasons, in the second part of the study, the analysis focused on the moderation effects of two internal variables, productivity and size of the board of directors, and an external variable, the environmental-sensitive industry status. The results proved a statistically significant influence of all moderating variables for the profitability indicator ROA, while for the current liquidity (CR), no statistical significance was found. Thus, the findings showed that if the status of the environmental-sensitive industry is taken into account as a moderating variable, the higher the environmental sensitive score, the stronger the positive correlation between waste information disclosure and financial performance. However, when board size, as well as productivity, were considered as moderating variables, with the increase in the board size or in the productivity level, the negative impact of *WMnID* on profitability is deepened, while liquidity proved not to be significant. As for the alternative metrics of financial performance, results revealed that a higher degree of waste management information disclosure will increase the profitability (the ROE and EPS) of the companies, while in the case of liquidity, the results are not conclusive; in the case of SOL the influence is rather negative, meaning that a higher level of *WMnID* will generate a decrease in companies’ solvability. Regarding the moderating effects, 2SLS analysis led to the following conclusions: the environmental-sensitive industry status positively affects the ROA, ROE, and EPS, revealing that in these manufacturing companies, a higher *WMnID* leads to an increased profitability, while no statistical significance was found in the case of liquidity indicators; the influence of board size is significantly negative on profitability and positive on solvability, and the influence of economic productivity is negatively significant for profitability indicators as well as for solvability, but not relevant in the case of current liquidity.

Nowadays, manufacturing companies are prone to invest in measures that increase resource efficiency and in strategies to optimize the renewable resource flow, such as waste-diminishing actions. Under such circumstances, this work results can be harnessed and shed light on more opportunities to enhance and improve the waste management information disclosure behavior of manufacturing companies, considering the following implications of the study.

### 6.1. Theoretical Implications

The study offers and opens new research routes for all those interested in the process of valorization and the reuse of resources from the perspective of the impact it has on the performance of manufacturing companies. In a framework provided by the financial reporting theories, researchers and other interested parties may be interested in the results of this study to track issues such as information asymmetry or agency cost management, as well as signals sent to various stakeholders. Mapping the waste flow connected to economic and financial performance objectives may offer insights into other hot issues raised from the examination of companies’ disclosure behavior. The theoretical implications of the study are many and various, especially from the perspective of new avenues of research and even innovation.

### 6.2. Governance, Management, and other Practical Implications

From the perspective of the practical implications, the findings of the study invites companies to reflect upon: (a) other possibilities for the development of new efficient waste management tools to permanently follow the correlations with the relevant financial performance indicators; (b) different options to design a personalized waste flow that allows managers to control all upstream and downstream costs and includes all sub-processes of resource utilization with other related additional costs; (c) possibilities to develop new governance strategies that allow the implementation of an efficient circuit of renewable resources and support the recovery of residual materials and fight for the reduction in toxic waste with an unfavorable impact on the environment and society; (d) options for rethinking managerial compensations and bonuses as well as employee salaries depending on the contributions to improving the company’s green behavior; € solid reasons for avoiding impression management, “*greenwashing*”, behavior to manipulate the waste management information disclosure, eager to obtain financing sources with lower costs by deceiving creditors; (f) possibilities of reducing the risk of investment projects and increasing the confidence of investors and creditors by increasing the quality of the waste management information disclosure; (g) development possibilities of new specific analysis proxies for companies in sensitive industries that adequately reflect the impact of waste and recyclable materials inputs on performance indicators; and (h) the implementation of new technologies to connect waste management information disclosure behavior to corporate performance and allow accurate predictions.

### 6.3. Regulatory Consequences

The results of the study may also have implications on the waste management regulatory process for companies in sensitive industries through the development or improvement of existing local regulations on waste recycling and the monitoring of waste reduction behavior by specific institutions. Knowing the extremely high pressure of the external environment on the companies that generate waste to track and implement policies to reduce them and eliminate the toxicity of the residues obtained from the production process and the complementary activities, they should be supported and helped in their endeavor, not an easy one, to become increasingly smarter and greener companies.

The main limits of the study can be found in the small sample of analyzed companies and in measuring the average degree of the waste management information disclosure index. Assigning scores after reading the information from the annual reports of the companies depends on the degree of detail of waste management information, and, of course, the subjectivity of the one who gives the grades can be a vulnerable point of this research. As well, the choice of moderating variables can be considered another issue that raises different approaches and interpretations. Thus, further research projects will focus on broadening the sample of companies, identifying other variables to test different moderation effects, and using decision tree models to predict waste information disclosure behavior related to corporate performance.

## Figures and Tables

**Table 1:** Description of the classification codes for selected manufacturing companies.

Number of Business Categories	CANE Code	Description
1	2014	Manufacture of other basic organic chemicals
2	2110	Manufacture of basic pharmaceutical products
3	2120	Manufacture of pharmaceutical products
4	2219	Manufacture of other rubber products
5	2221	Manufacture of plastic plates, foils, tubes, and profiles
6	2229	Manufacture of other plastic products
7	2332	Manufacture of bricks, tiles, and other products of burnt clay construction
8	2361	Manufacture of concrete products for construction
9	2391	Manufacture of abrasive products
10	2442	Aluminum metallurgy
11	2599	Manufacture of other fabricated metal products n.e.c.
12	2651	Manufacture of instruments and devices for measurement, verification, control, navigation
13	2711	Manufacture of electric motors, generators, and transformers
14	2712	Manufacture of electricity distribution and control equipment
15	2731	Manufacture of fiber optic cables
16	2751	Manufacture of household appliances
17	2811	Manufacture of engines and turbines (except for aircraft, motor vehicles, and motorcycles)
18	2814	Manufacture of taps and fittings
19	2830	Manufacture of machinery and equipment for agriculture and forestry
20	2892	Manufacture of machinery for mining and construction
21	2932	Manufacture of other parts and accessories for motor vehicles and motor vehicles

**Table 2:** Variables definition.

Symbol	Name	Measurement	Type
ROE	Return on equity	Net profit/equity	Financial performance (profitability indicator), explained variable
ROA	Return on assets	Net profit/total assets	Financial performance (profitability indicator), explained variable
EPS	Earnings per share	Net profit/average number of ordinary shares outstanding for the reported year	Financial performance (profitability indicator), explained variable
SOL	Solvability	Total assets/total liabilities	Financial performance (liquidity indicator), explained variable
CR	Current ratio	Current assets/current liabilities	Financial performance (liquidity indicator), explained variable
*WMnID*	Waste Management Information Disclosure	Average disclosure score on waste management information	Explanatory/independent variable
PRD	Average annual productivity	Net sales/average number of employees	Moderating variable
BS	Board size	Number of managers on the board	Moderating variable
IND	Industry	Score 1 was assigned if the company belongs to an environmentally sensitive industry and 0 otherwise	Moderating variable
BIG4	Audit report	Score 1 was assigned if the annual financial statements are audited by one of the big four companies and 0 otherwise	Control variable
Size	Turnover	Total net sales expressed in billions of lei	Control variable
EPLY	Employees	The average number of employees expressed in thousand of persons	Control variable
LEV	Leverage	Total debts/total equity	Control variable

**Table 3:** Descriptive statistics results.

	ROA	ROE	SOL	CR	EPS	Envir_Sensitive Status	*WMnID*	Turnover	No of Employees	Board_Size	Big4	PRD	LEV
Mean	0.023	0.027	3.771	2.538	−0.216	0.643	1.164	261,000,000	514.986	4.579	0.286	434,437.600	−0.053
Median	0.019	0.036	2.973	1.998	0.045	1.000	1.000	95,694,343	335.500	5.000	0.000	280,952.500	0.434
Maximum	1.256	1.812	30.018	13.803	14.971	1.000	3.000	3,300,000,000	2549.000	13.000	1.000	2,794,691.000	6.385
Minimum	−0.814	−3.700	0.308	0.051	−33.596	0.000	0.000	542,415	1.000	1.000	0.000	59,295.170	−26.570
Std. Dev.	0.162	0.414	3.504	2.075	3.833	0.481	1.215	521,000,000	579.683	1.662	0.453	388,007.400	3.085
Skewness	2.255	−4.547	4.768	2.160	−6.027	−0.596	0.552	3.921	2.016	1.413	0.949	2.571	−6.305
Kurtosis	30.510	51.221	33.166	9.461	54.134	1.356	1.720	18.947	6.809	9.665	1.900	12.929	49.684
Jarque-Bera	4533.434	14,046.520	5838.881	352.327	16,100.100	24.071	16.672	1842.109	179.487	305.721	28.058	729.356	13,640.820
Probability	0.000	0.000	0.000	0.000	0.000	0.000	0.000	0.000	0.000	0.000	0.000	0.000	0.000
Observations	140	140	140	140	140	140	140	140	140	140	140	140	140

**Table 4:** Correlation matrix.

Correlation Probability	ROA	ROE	SOL	CR	EPS	*WMnID*	Env_Sens	Board Size	PRD	No of Empl.	Big4	LEV	Turnover
ROA	1.00												
	-----												
ROE	0.56	1.00											
	0.00	-----											
SOL	0.08	0.05	1.00										
	0.35	0.56	-----										
CR	0.17	0.04	0.38	1.00									
	0.05	0.65	0.00	-----									
EPS	0.55	0.23	0.08	−0.06	1.00								
	0.00	0.01	0.36	0.50	-----								
*WMnID*	0.15	0.05	−0.19	−0.05	0.08	1.00							
	0.0681 *	0.60	0.0213 **	0.57	0.36	-----							
Env_sens	0.07	−0.08	0.17	0.15	0.09	0.18	1.00						
	0.38	0.35	0.0419 **	0.0716 **	0.29	0.0386 **	-----						
Board size	0.19	0.13	−0.02	0.06	0.18	0.36	0.17	1.00					
	0.0261 **	0.12	0.84	0.45	0.0354 **	0.0000 ***	0.0442 **	-----					
PRD	0.11	0.09	−0.06	0.01	0.02	0.19	−0.06	0.09	1.00				
	0.19	0.26	0.48	0.90	0.81	0.0257 **	0.47	0.28	-----				
No of empl.	0.05	−0.01	−0.26	−0.12	0.03	0.58	−0.02	0.47	0.17	1.00			
	0.58	0.95	0.0024 **	0.17	0.69	0.0000 ***	0.79	0.0000 ***	0.0512 *	-----			
Big4	0.15	0.10	−0.02	0.16	0.05	0.25	0.14	0.35	0.39	0.17	1.00		
	0.0786 **	0.24	0.83	0.0579 *	0.53	0.0025 ***	0.10	0.0000 ***	0.0000 ***	0.0487 **	-----		
LEV	0.19	0.20	0.11	0.09	0.08	0.18	0.13	0.09	0.05	0.16	0.13	1.00	
	0.0228 **	0.0158 **	0.21	0.29	0.33	0.0308 **	0.14	0.28	0.54	0.0554 **	0.12	-----	
Turnover	0.07	0.06	−0.18	−0.01	0.04	0.41	0.04	0.54	0.58	0.78	0.34	0.10	1.00
	0.40	0.52	0.0304 **	0.94	0.64	0.0000 ***	0.60	0.0000 ***	0.0000 ***	0.0000 ***	0.0000 ***	0.23	-----

*, **, and *** represent 10%, 5%, and 1% significance level, respectively.

**Table 5:** Variance inflationary factor (VIF) results.

Variable	Centered VIF
LEV	1.066001
*WMnID*	1.696403
Board size	1.545459
Turnover	3.172071
Average no. of employees	3.640687
BIG4	1.302361
Environmental-sensitive status	1.114015
Average	1.933857

**Table 6:** The moderating effects regression results.

	ROA				CR			
	M1	M2	M3	M4	M5	M6	M7	M8
*WMnID* Environ_sensitive_status	0.0079 ***	−0.011 ***	0.068 ***	0.025 ***	0.012	0.022 ***	−0.078	0.003
Board size	−0.0017	−0.0043	0.013 ***	−0.006	−0.108 *	−0.11 ***	−0.158	−0.105 **
PRD				0.198 ***				−0.26
Moderating effects								
Envir_sensitive × *WMnID*		0.0226 ***				0.033		
Board size × *WMnID*			−0.011 ***				0.017	
PRD × *WMnID*				−0.049 ***				−0.03
Control variables								
Turnover	0.09 **	0.129 **	0.105 **		0.146	0.249	0.065 ***	
No of employees	0.035 *	0.008	0.02		0.169 *	0.311 ***	0.199 **	
LEV	0.004 **	0.004 **	0.0044 **	0.0038 *	−0.04 ***		−0.04 *	-0.038 *
Big4						-0.04***		
Constant	−0.019 **	−0.0012	−0.08 ***	−0.004	2.89 ***	2.77 ***	3.129 ***	3.15 ***
Obsevations	140	140	140	140	140	140	140	140
F-test	20.53 ***	20.59 ***	21.52 **	26.27 **	18.62 ***	20.39 **	18.01 **	17.01 **
S.E. of Reg.	0.14	0.14	0.14	0.14	1.16	1.17	1.16	1.14
R^2^	0.859	0.86	0.87	0.88	0.84	0.86	0.85	0.83
Jarque-Bera test	3.19 (0.20)	2.28 (0.31)	3.22 (0.199)	4.54 (0.10)	5.97 (0.05)	5.86 (0.05)	5.87 (0.05)	5.74 (0.05)
Redundant Fixed Effects- Likelihood Ratio test prob.	0.036	0.039	0.032	0.037	0.00	0.00	0.00	0.00
Hausman test prob.	0.03	0.06	0.05	0.013	0.02	0.02	0.03	0.04
Residual Cross-Section Dependence Test Breusch–Pagan LM test	522.41 (0.00)	509.91 (0.00)	497.95 (0.00)	496.96 (0.00)	580.34 (0.00)	585.74 (0.00)	573.82 (0.00)	572.42 (0.00)
Panel Cross Section Heteroskedasticity LR test	275.59 (0.00)	149.41 (0.00)	271.03 (0.00)	283.22 (0.00)	244.38 (0.00)	242.92 (0.00)	255.30 (0.00)	243.19 (0.00)

Note: ***, **, * mean statistically significant at 1%, 5%, and 10%; () represents the probability.

**Table 7:** Endogeneity test of the moderating effects regression results using 2SLS estimation.

	ROE				SOL				EPS			
	Endogeneity Problem (2SLS) + Alternative Measures of Financial Performance											
	M1′	M2′	M3′	M4′	M5′	M6′	M7′	M8′	M9′	M10′	M11′	M12′
*WMnID*	0.028 ***	−0.0498 ***	0.129 ***	0.055 ***	−0.393 ***	−0.37 ***	−1.03 ***	−0.09	0.033 ***	−0.150 ***	0.321 ***	0.292 ***
Board size	−0.034 ***	−0.0167 ***	0.008	−0.03 ***	−0.146*	−0.025	−0.379 ***	−0.108 **	−0.039 *	−0.012	0.057	−0.024 ***
PRD				0.23 ***				0.97 ***				1.30 ***
Moderating effects												
Envir_sensitive × *WMnID*		0.0088 ***				0.148				0.194 ***		
Board size × *WMnID*			−0.020 ***				0.126 ***				−0.055 ***	
PRD × *WMnID*				−0.06 ***				−0.43 ***				−0.389 ***
Control variables												
Turnover	0.242 ***	0.129 **	0.250 ***		0.292 ***	−0.043	0.178 ***		2.667 ***	2.927 ***	3.23 ***	
No of employees	−0.067	0.008	−0.07		0.915	0.63 *	0.95		−0.38 ***	−0.439 ***	−0.39 ***	
LEV	−0.0007	0.003	0.00056	0.01	0.024	−0.0099	0.019	−0.021 *	0.01 *	0.01 *	0.0098 *	−0.002
Constant	0.12 ***	0.10	−0.08 ***	0.057	4.44 ***	3.97 ***	5.51 ***	4.27 ***	−0.434 ***	−0.55 ***	−0.977 ***	−0.64 ***
R^2^	0.854	0.843	0.864	0.89	0.80	0.97	0.80	0.89	0.83	0.847	0.824	0.55

Note: ***, **, * mean statistically significant at 1%, 5%, and 10%; () represents the probability.

## Data Availability

The analyzed data during the current study are available from the corresponding author upon reasonable request.
